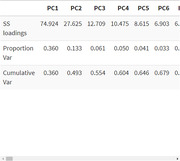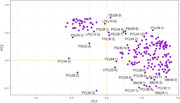# Principal Component Analysis discriminates lipid metabolites in the plasma of individuals with MCI, Alzheimer's, and Down Syndrome

**DOI:** 10.1002/alz.092268

**Published:** 2025-01-09

**Authors:** Tamires Alves Sarno, Cícero Augusto Costa Pereira, Leda Leme Talib, Wagner Farid Gattaz, Orestes Vicente Forlenza

**Affiliations:** ^1^ Laboratory of Neurosciences, Institute of Psychiatry, Faculty of Medicine, University of São Paulo, Sao Paulo Brazil; ^2^ Institute of Psychiatry, Faculty of Medicine, University of São Paulo., São Paulo Brazil; ^3^ Laboratório de Neurociências ‐ LIM 27, Sao Paulo Brazil; ^4^ University of São Paulo, São Paulo, Brazil, Sao Paulo, São Paulo Brazil; ^5^ Laboratory of Neuroscience (LIM‐27), Department and Institute of Psychiatry, Faculty of Medicine, University of São Paulo, São Paulo, São Paulo Brazil

## Abstract

**Background:**

There is evidence indicating that disruptions in lipid metabolism are implicated in the pathophysiology of Alzheimer's disease (AD), with systemic repercussions that can be identified in peripheral blood. Recent studies conducted by our group have identified abnormalities in lipid metabolism among patients with mild cognitive impairment (MCI) and dementia (probable AD), through the investigation of a specific panel of lipid metabolites in plasma. Although much remains to be elucidated about the complex interaction between disturbances in lipid metabolites and the pathogenesis of AD, this promising research area offers exciting opportunities for the development of new strategies for disease diagnosis, treatment, and prevention.

**Method:**

Blood plasma samples were collected from the following groups: individuals with Alzheimer's disease (AD, n=17), individuals with Mild Cognitive Impairment (MCI) showing pathological AD signature (n=17), individuals with MCI without pathological AD signature (n=17), individuals with Down syndrome (n=17), elderly (n=17), and young (n=17) cognitively healthy individuals. We used liquid chromatography coupled with mass spectrometry (LC‐MS/MS) for lipidomic analysis.

**Result:**

A total of 29 lipids were identified by principal component analysis (PCA) from a dataset consisting of 208 lipids. The lipid modules (attached image) composed of lysophosphatidylcholine, lysophosphatidylethanolamine, phosphatidylcholine, phosphatidylethanolamine, phosphatidylserine, phosphatidylglycerol and sphingomyelin are differentially expressed in the samples. PCA1 (74.92%) and PCA2 (27.6%) account for the majority of variance in the sample (attached table).

**Conclusion:**

Our preliminary findings suggest that the use of PCA analysis may aid in discriminating differently expressed lipids in the plasma of individuals with dementia syndromes